# Anti-inflammatory activity of edible oyster mushroom is mediated through the inhibition of NF-κB and AP-1 signaling

**DOI:** 10.1186/1475-2891-10-52

**Published:** 2011-05-16

**Authors:** Andrej Jedinak, Shailesh Dudhgaonkar, Qing-li Wu, James Simon, Daniel Sliva

**Affiliations:** 1 Cancer Research Laboratory, Methodist Research Institute, Indiana University Health, 1800 N Capitol Ave, E504, Indianapolis, IN 46202, USA; 2 New Use Agriculture and Natural Plant Products Program, Department of Plant Biology and Plant Pathology, Rutgers, The State University of New Jersey, New Brunswick, NJ 08901, USA; 3 Department of Medicine, School of Medicine, Indiana University, Indianapolis, IN, USA; 4 Indiana University Simon Cancer Center, School of Medicine, Indiana University, Indianapolis, IN, USA

## Abstract

**Background:**

Mushrooms are well recognized for their culinary properties as well as for their potency to enhance immune response. In the present study, we evaluated anti-inflammatory properties of an edible oyster mushroom (*Pleurotus ostreatus*) *in vitro *and *in vivo*.

**Methods:**

RAW264.7 murine macrophage cell line and murine splenocytes were incubated with the oyster mushroom concentrate (OMC, 0-100 μg/ml) in the absence or presence of lipopolysacharide (LPS) or concanavalin A (ConA), respectively. Cell proliferation was determined by MTT assay. Expression of cytokines and proteins was measured by ELISA assay and Western blot analysis, respectively. DNA-binding activity was assayed by the gel-shift analysis. Inflammation in mice was induced by intraperitoneal injection of LPS.

**Results:**

OMC suppressed LPS-induced secretion of tumor necrosis factor-α (TNF-α, interleukin-6 (IL-6), and IL-12p40 from RAW264.7 macrophages. OMC inhibited LPS-induced production of prostaglandin E2 (PGE_2_) and nitric oxide (NO) through the down-regulation of expression of COX-2 and iNOS, respectively. OMC also inhibited LPS-dependent DNA-binding activity of AP-1 and NF-κB in RAW264.7 cells. Oral administration of OMC markedly suppressed secretion of TNF-α and IL-6 in mice challenged with LPS *in vivo*. Anti-inflammatory activity of OMC was confirmed by the inhibition of proliferation and secretion of interferon-γ (IFN-γ), IL-2, and IL-6 from concanavalin A (ConA)-stimulated mouse splenocytes.

**Conclusions:**

Our study suggests that oyster mushroom possesses anti-inflammatory activities and could be considered a dietary agent against inflammation. The health benefits of the oyster mushroom warrant further clinical studies.

## Background

Systemic inflammation has been linked to the pathogenesis of a variety of diseases including endotoxemia and sepsis [1]. Pro-inflammatory cells, mainly macrophages, monocytes, or other host cells, respond to invading pathogens by releasing pro-inflammatory mediators, including tumor necrosis factor-α (TNF-α), interleukin-6 (IL-6), IL-12, cyclooxygenase-2 (COX-2) and nitric oxide (NO) [2-5]. Mechanistically, lipopolysaccharide (LPS), a constituent of the cell wall of gram-negative bacteria, interacts with toll-like receptor 4 (TLR4), which is expressed on pro-inflammatory cells [6,7]. The interaction between LPS and the TLR4 receptor complex results in the activation of intracellular signaling through MyD88 and TRIF pathways leading to the activation of transcription factors NF-κB and AP-1 and the expression of TNF-α and IL-6 [8,9]. LPS also induces expression of IL-12 in macrophages through the NF-κB pathway [10,11], and the AP-1 binding site was identified in the promoter region of IL-12p40 [12]. Moreover, LPS-induced expression of COX-2 and inducible nitric oxide synthase (iNOS) is also controlled through NF-κB and AP-1 [13], and LPS-induced expression of iNOS and IL-6 require phosphorylation of STAT3 [14,15]. Therefore, the suppression of the release of pro-inflammatory mediators, through the modulation of intracellular signaling in immune cells, could be used for the treatment or prevention of endotoxemia and sepsis. One of the possible strategies to suppress systemic inflammation is the employment of natural compounds such as botanicals, bioactive food components, or functional foods.

Mushrooms have been used, as a source of food and for their medicinal (generally anticancer) properties, since ancient times [16]. A number of biologically active compounds, including polysaccharides, vitamins, terpenes, steroids, amino acids, and trace elements, have been identified in different mushroom species [17]. Polysaccharides, mainly α- or β-glucans, protein-bound polysaccharides, or glycoproteins, demonstrated immunomodulatory activities through (i) increased production of cytokines (IL-10, IL-12p70 and IL-12p40) by dendritic cells (DC), (ii) activation of natural killer (NK) cells, and (iii) increased production of TNF-α, IL-1, IL-6, IL-8, IL-12p40, and NO, and expression of iNOS by macrophages [18]. The majority of these studies were performed with medicinal (*Ganoderma lucidum*, *Phellinus linteus*) or edible (*Agaricus blazei, Grifola frondosa*) mushrooms [18]. As recently demonstrated, an edible white button mushroom (*Agaricus bisporus*) enhanced NK cell activity in mice through the increased production of IFN-γ which induced maturation of dendritic cells, and TNF-α, which increased production of IL-12 [19,20].

Oyster mushrooms (*Pleurotus *species) belong to the world of consumed mushrooms that, in addition to their nutritional value, demonstrate health-promoting (antioxidant, anti-atherosclerotic, anticancer and immunomodulatory) effects [21-24]. Glycosphingolipid, isolated from *Pleurotus eryngii*, induced secretion of IFN-γ and IL-4 from T-cells [25], whereas β-glucan demonstrated an anti-inflammatory response in a model of acute colitis in rats when isolated from *Pleurotus pulmonarius*, and, when isolated from *Pleurotus ostreatus*, inhibited leukocyte migration to acetic acid-injured tissues [26,27]. An extract from *Pleurotus florida *suppressed inflammation, as demonstrated by the decrease in paw thickness in carrageen-induced acute inflammation and formalin-induced chronic inflammation [28]. However, the molecular mechanisms responsible for the anti-inflammatory properties of the oyster mushroom were not addressed. In the present study, we examined the anti-inflammatory effects of the oyster mushroom on LPS-stimulated RAW264.7 macrophages, isolated murine splenocytes, and in mice challenged with LPS.

## Methods

### Materials

Oyster mushrooms were provided by Forest mushrooms, Inc. (Saint Joseph, MN, USA). Oyster mushrooms were grown at 17-18°C, relative humidity 90-95% and 850 ppm CO_2 _with diffuse daylight during the day and no artificial light at night. Oyster mushrooms were harvested at 5 days of age and shipped O/N at 4°C to the laboratory for the further processing. Lipopolysaccharide (LPS) and concanavalin A were purchased from Sigma (St. Louis, MO, USA), and heparin was purchased from Abraxis Pharmaceutical Products (Schaumburg, IL, USA).

### Preparation of Oyster mushroom concentrate (OMC)

Fresh oyster mushrooms were ground and then lyophilized by FreeZone^® ^4.5 Liter Freeze Dry Systems, and the dry biomass was stored at 4°C. The ground materials were then re-suspended in distilled water at the concentration 5 mg/ml and incubated overnight at 4°C. The suspension was centrifuged at 7000 rpm (5200 g) for 10 minutes, and the supernatant was sterilized through a 0.22 μm filter. This water-soluble lyophilized oyster mushroom was labeled oyster mushroom concentrate (OMC), and its concentration corresponded to the original lyophilized oyster mushroom. An Osmomat μ OSMETTE™ (Precision Systems Inc, Natick, MA, USA) was used to determine osmolarity of the OMC extract (26 mOsm/kg) and Orion 2-Star Benchtop pH meter (Water Analysis Instruments, Beverly, MA, USA) for pH determination (pH = 6.2).

### Cells

The murine macrophage cell line (RAW264.7) was obtained from ATCC (Manassas, VA, USA). The cells were maintained in Dulbecco's modified Eagle's medium (DMEM) supplemented with 10% fetal bovine serum (FBS), 100 units/ml of penicillin, and 100 μg/ml of streptomycin (all from Gibco, Grand Island, NY, USA) in a humidified atmosphere at 37°C in 5% CO_2_.

### Cell proliferation-viability

RAW264.7 cell were treated with OMC (0-100 μg/ml) at indicated times and cell proliferation-viability was evaluated by MTT assay as previously described [29].

### Expression of pro-inflammatory mediators in RAW264.7 cells

RAW264.7 cells were pretreated with OMC (0-100 μg/ml) for 24 hours, followed by stimulation with 1 μg/ml of LPS or TNF-α 100 ng/ml (R&D Systems, Minneapolis, MN, USA) for an additional 24 hours in medium without FBS and antibiotics. Then the medium was collected and centrifuged to remove debris. Secretion of TNF-α, IL-6, IL-12p40 (Biolegend, San Diego, CA, USA), and PGE_2 _(R&D Systems, Minneapolis, MN, USA) was determined by ELISA; the release of NO was determined by Griess reagent (Sigma, St. Louis, MO, USA) as previously described [29].

### Western blot analysis

RAW264.7 cells were pretreated with OMC (0-100 μg/ml) for 24 hours followed by incubation with LPS (1 μg/ml) for an additional 30 minutes (STAT3) or 24 hours (COX-2, iNOS). Whole cell extracts were prepared and subjected to Western blot analysis with anti-COX-2, anti-iNOS, anti-phospho-STAT3, and anti-STAT-3 antibodies (Santa Cruz Biotechnology, Santa Cruz, CA, USA), respectively, as we previously described [29]. The equivalent amount of proteins was verified by reprobing the blot with anti-actin antibody (Santa Cruz Biotechnology, Santa Cruz, CA, USA). The expression of each protein was detected by the ECL Western blotting detection system (Amersham Biosciences, Buckinghamshire, UK).

### Electrophoretic mobility shift assay (EMSA)

EMSA for AP-1 and NF-κB was performed with nuclear extracts isolated from RAW264.7 cells pretreated with OMC (0-100 μg/ml) and stimulated with LPS (1 μg/ml) at indicated times as previously described [30]. Briefly, nuclear extracts were incubated with ^32^P-labelled NF-B or AP-1 oligonucleotide probes, and DNA-binding was detected by autoradiography after the separation of DNA-nuclear protein complexes on PAGE gel as previously described [29]. Oligonucleotide probes containing consensus sequences for AP-1 and NF-κB binding sites were purchased from Promega (Madison, WI, USA).

### Densitometric analysis

Autoradiograms of the Western blots and gel shifts were scanned with HP scanjet 5470c scanner. The optical densities on the films were quantified and analyzed with the UN-SCAN-IT software (Silk Scientific, Orem, UT). The ratios of COX-2/β-actin, iNOS/β-actin and phospho-STAT3/STAT3 were calculated by standardizing the ratios of each control to the unit value.

### Animal experiments

Eight- to ten-week-old female Balb/C mice were obtained from the Jackson Laboratory, (Bar Harbor, ME, USA) and were maintained at the Methodist Research Institute Animal Facility which is accredited and certified by USDA with PHS assurance (A3772-01). Animal experiments were fully approved by the Methodist Research Institute Animal Research Committee (ARC 2007-17). All animals were housed in plastic cages (4 mice/cage) with free access to drinking water and NIH#31M pellet diet ad libitum. After one week of acclimatization, the mice were randomly divided into experimental and control groups. To avoid the interference of food in the absorption of the treatment agent, the mice were starved for 3 hours before the experiment started. In the next step OMC (1000 mg/kg of body weight) or distilled water was administered by intragastrical gavage. The concentration of 1000 mg/kg of the oyster mushroom extract was previously used for the inhibition of acute and chronic inflammation in mice [28]. After one hour, the mice received intraperitoneal injection of LPS (0.1 mg/kg of body weight). After an additional 90 minutes, the blood was withdrawn by the retroorbital venipuncture, and plasma was isolated by centrifugation. All animals were euthanized by CO_2_asphyxiation. Plasma TNF-α and IL-6 levels were determined by ELISA according to the manufacturer's instructions (Biolegend, San Diego, CA, USA).

### Proliferation of splenocytes

Balb/C mice were sacrificed and their spleens were removed and homogenized aseptically. 2.5 × 10^4 ^spleen cells were cultured in 96-well plates in triplicates in complete medium consisting of RPMI 1640 supplemented with 10% FBS, 1% [+]-glutamine, 100 units/ml of penicillin, and 100 μg/ml of streptomycin (all from Gibco, Grand Island, NY, USA). The cells were cultured in the presence of concanavalin A (5 μg/ml) and OMC (0-100 μg/ml) for 72 hours, followed by the addition of cell proliferation reagent WST-1 (Roche Applied Science, Indianapolis, IN, USA) for another 3 hours. After that, the microplate was read at 450 nm by a Versamax microplate reader (Molecular Devices, Sunnyvale, CA, USA).

### Cytokine production in splenocytes

1 × 10^5 ^spleen cells were cultured in duplicate in 24-well plates containing RPMI 1640 medium supplemented with 10% FBS, 1% [+]-glutamine, 100 units/ml of penicillin and 100 μg/ml of streptomycin (all from Gibco, Grand Island, NY, USA). The cells were cultured in the presence of concanavalin A (5 μg/ml) and OMC (0-100 μg/ml) for 72 hours, and the medium was collected and centrifuged to remove debris. Secretion of IFN-γ, IL-2 and IL-6 was determined by ELISA (Biolegend, San Diego, CA, USA).

### Analysis of glucans in lyophilized oyster mushrooms and OMC

α- and β-glucans in lyophilized oyster mushrooms and OMC were determined by acidic and enzymatic hydrolysis using the Mushroom and Yeast beta-glucan assay according to manufacturer's protocol (Megazyme, Wicklow, Ireland).

### LC/UV/MS analysis

Lyophilized oyster mushrooms were extracted with water and analyzed by liquid chromatography/ultraviolet spectrometry/mass spectrometry (LC/UV/MS). The LC/UV/MS methods for natural compounds were developed and evaluated by the New Use Agriculture and Natural Products Program (NUANPP), and performed on an Agilent 1100 Series LC/UV/MSD system equipped with a quaternary pump, diode array detector (DAD), thermostatted column compartment, degasser, MSD ion trap with an electrospray ion source (ESI) and HP ChemStation software, Bruker Daltonics 4.2 and DataAnalysis 4.2 software. HPLC separation was performed with the mobile phase consisting of solvent water and methanol in gradient. The wavelength of UV detection was set at 210 and 254 nm. Column compartment was set at 25°C. The flow rate was 1.0 mL/min. The electrospray ion mass spectrometer (ESI-MS) was operated under positive ion and optimized collision energy level of 60%, scanned from *m/z *100 to 600. ESI was conducted using a needle voltage of 3.5 kV. High-purity nitrogen (99.999%) was used as dry gas, and a nebulizer was used at a flow rate of 12 L/min; capillary temperature was 350°C. Helium, at 60 psi, was used as the collision gas. The ESI interface and mass spectrometer parameters were optimized to obtain maximum sensitivity.

### Statistical Analysis

Data are mean ± S.D and were analyzed by ANOVA or Student's t-test. Results were considered significant if *P *≤ 0.05.

## Results

### OMC suppresses LPS-dependent induction of cytokines and inflammatory mediators in macrophages

**Figure 1:**
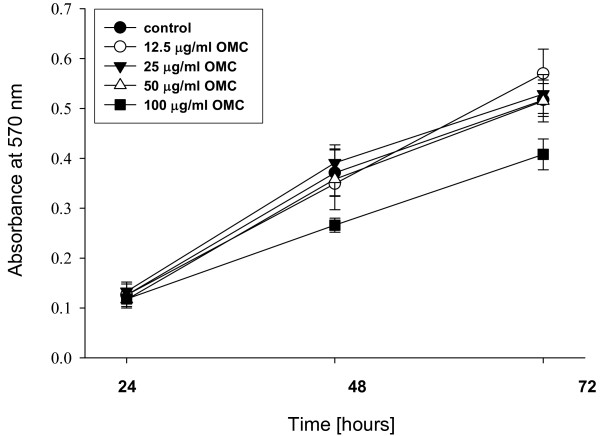
**Effect of OMC on viability of macrophages**. RAW264.7 cells were treated with OMC (0-100 μg/ml) for 24, 48 and 72 hours and cell proliferation-viability determined as described in *Materials and Methods*. The data are means ± S.D., *n *= 3-4.

To assess whether OMC possesses anti-inflammatory activity *in vitro*, we first determined whether OMC affects the production of pro-inflammatory cytokines in macrophages exposed to inflammatory stimuli. As seen in Table 1 OMC treatment significantly suppressed LPS-dependent production of TNF-α, IL-6, and IL-12, respectively, in a dose response manner. This effect was not caused by the cytotoxicity of OMC because OMC did not affect viability of RAW264.7 cells; the only slight inhibition of cell proliferation (viability) was observed after 48 and 72 hours at the highest concentration (100 μg/ml) of OMC treatment (Figure 1). Therefore, the inhibition of production of TNF-α, IL-6, and IL-12 by 62%, 93% and 64%, respectively, at 100 μg/ml of OMC (Table 1) is mediated by the anti-inflammatory activity of OMC.

**Table 1:** OMC Suppresses LPS-induced inflammatory response in RAW264.7 macrophages.

	TNF-α [ng/ml]	IL-6 [ng/ml]	IL-12 [pg/ml]
Control	3.96 ± 0.15	1.73 ± 0.07	1.65 ± 1.51
LPS + 0 μg/ml OMC	15.35 ± 1.41†	16.12 ± 0.12†	108.75 ± 3.37†
LPS + 12.5 μg/ml OMC	12.99 ± 0.73*	9.54 ± 0.13*	68.01 ± 3.51*
LPS + 25 μg/ml OMC	11.59 ± 0.30*	6.20 ± 0.63*	58.55 ± 3.91*
LPS + 50 μg/ml OMC	9.04 ± 0.15*	4.18 ± 0.03*	47.41 ± 3.60*
LPS + 100 μg/ml OMC	5.87 ± 0.11*	1.16 ± 0.21*	39.18 ± 5.18*

The data are means ± S.D. of two independent experiments, repeated minimally twice.

† p < 0.05 ANOVA, LPS vs control

* p < 0.05 ANOVA, LPS vs OMC

**Figure 2:**
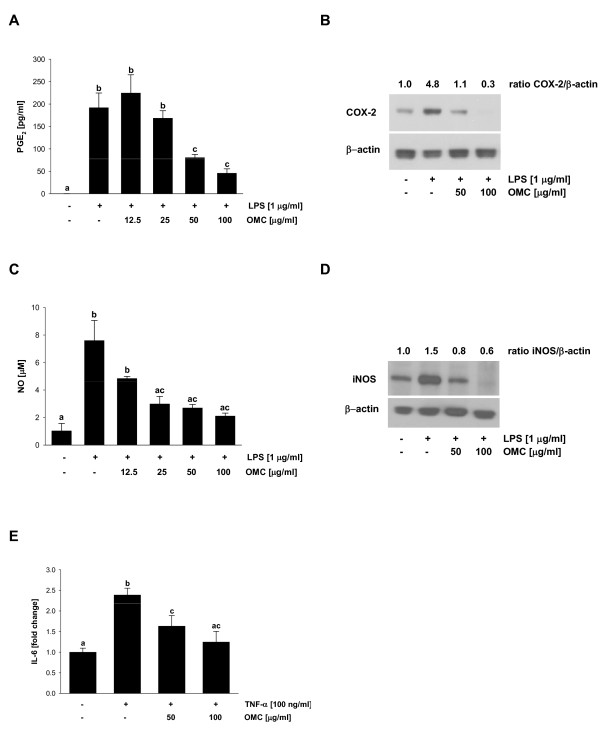
**Effect of OMC on LPS-induced PGE2 and NO secretion and COX-2 and iNOS expression in RAW264.7 cells**. (A) PGE_2 _and (C) NO secretion were determined in cell culture media from RAW264.7 cells treated with OMC and LPS as described in *Materials and Methods*. The data are means ± S.D. of two independent experiments, repeated minimally twice. Means without a common letter differ, *P *< 0.05. Expression of (B) COX-2 and (D) iNOS were determined in whole cell lysates from RAW264.7 cells treated with OMC and LPS as described in *Materials and Methods*. The equal protein loading was verified with anti-β-actin antibody. The results are representative of three separate experiments. (E) Secretion of IL-6 was determined in cell culture media from RAW264.7 cells treated with OMC and TNF-α as described in *Materials and Methods*. The data are means ± S.D. of two independent experiments, repeated minimally twice. Means without a common letter differ, *P *< 0.05.

Next, we evaluated whether OMC suppresses LPS-dependent production of inflammatory mediators PGE_2 _and NO. OMC inhibited production of PGE_2 _in RAW264.7 cells in a dose response manner (Figure 2A). To determine if this effect is caused by the inhibition of COX-2, we evaluated the expression of COX-2 by Western blot analysis. Therefore, LPS-induced expression of COX-2 in RAW 264.7 cells was markedly suppressed by the OMC treatment (Figure 2B). Moreover, OMC suppressed LPS-dependent production of NO in RAW264.7 cells (Figure 2C). As expected, this effect was directly linked to the down-regulation of iNOS by OMC in LPS-challenged macrophages (Figure 2D). To evaluate whether the effect of OMC is anti-inflammatory and is not mediated by the inhibition of endotoxin binding to TLR4 or inhibition of TLR4 signaling, we stimulated RAW264.7 cells with TNF-α. As seen in Figure 2E, TNF-α-dependent induction of IL-6 was markedly suppressed by the OMC treatment. Each of these screens showed that OMC suppressed proinflammatory response, LPS dependent production of cytokines and inflammatory mediators, in RAW264.7 macrophages.

### Effect of OMC on the LPS-dependent induction of transcription activity of AP-1, NF-κB, and STAT3

**Figure 3:**
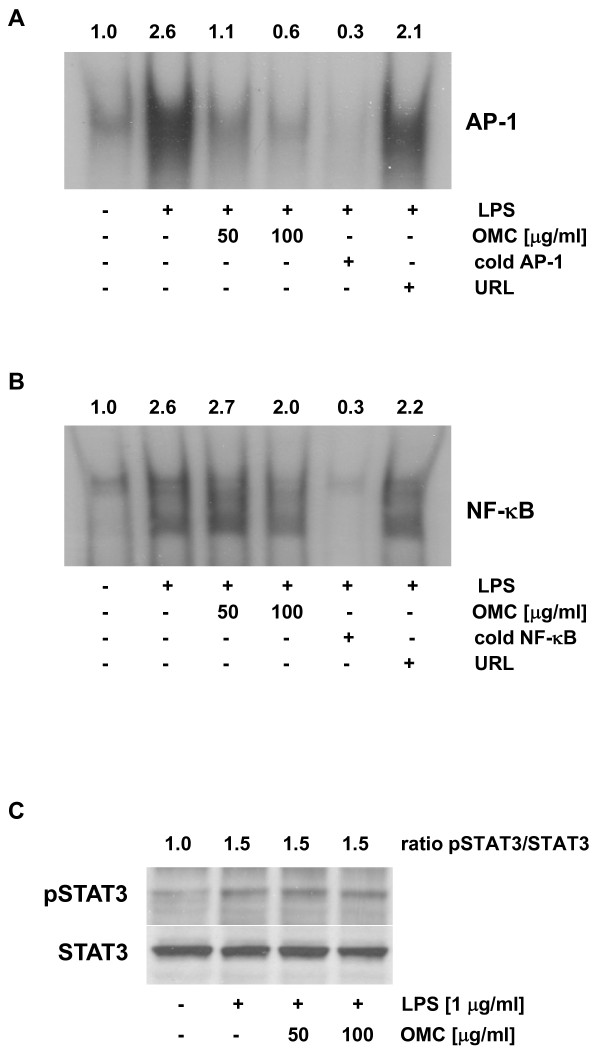
**Effect of OMC on LPS-dependent activation of AP-1, NF-κB and STAT3 in RAW264.7 cells**. (A) AP-1 and (B) NF-κB DNA-binding activity was determined by gel shift analysis in nuclear extracts isolated from RAW264.7 cells pretreated with OMC (0, 50 100 μg/ml) for 24 hours followed by the stimulation with LPS (1 μg/ml) for an additional 30 minutes. Nuclear extracts were subjected to EMSA with a [^32^P]-labeled AP-1 or [^32^P]-labeled NF-κB probe as described in *Materials and Methods*. The specificity of DNA-binding was confirmed by competitive gel shift with cold AP-1, NF-κB, or unrelated DNA (URL). (C) STAT3 activity was evaluated in whole cell extracts treated with OMC for 24 hours and LPS for 30 minutes by Western blot analysis with anti-phospho-STAT3 antibody. The equal protein loading was verified with anti-STAT3 antibody. The results are representative of three separate experiments.

As indicated, an inflammatory response in macrophages can be mediated through COX-2 and iNOS. The expression of COX-2 and iNOS is regulated by transcription factors AP-1, NF-κB, and STAT3 [15,30,31]. Nuclear extracts were prepared and EMSA with AP-1 and NF-κB performed. We observed that LPS-dependent induction of AP-1 binding activity was markedly suppressed by OMC in a dose-response manner (Figure 3A). On the other hand, LPS-dependent activation of NF-κB was only slightly reduced at 100 μg/ml of OMC (Figure 3B). Moreover, LPS also stimulated activity of STAT3, as demonstrated by the increased phosphorylation of STAT3 by Western blot analysis (Figure 3C). However, this LPS-dependent activation of STAT3 was not affected by the OMC (Figure 3C). In sum, the inhibition of an inflammatory response in macrophages is mediated by suppression of transcription activity of AP-1 and NF-κB.

### Effect of OMC on systemic inflammation in mice

**Figure 4:**
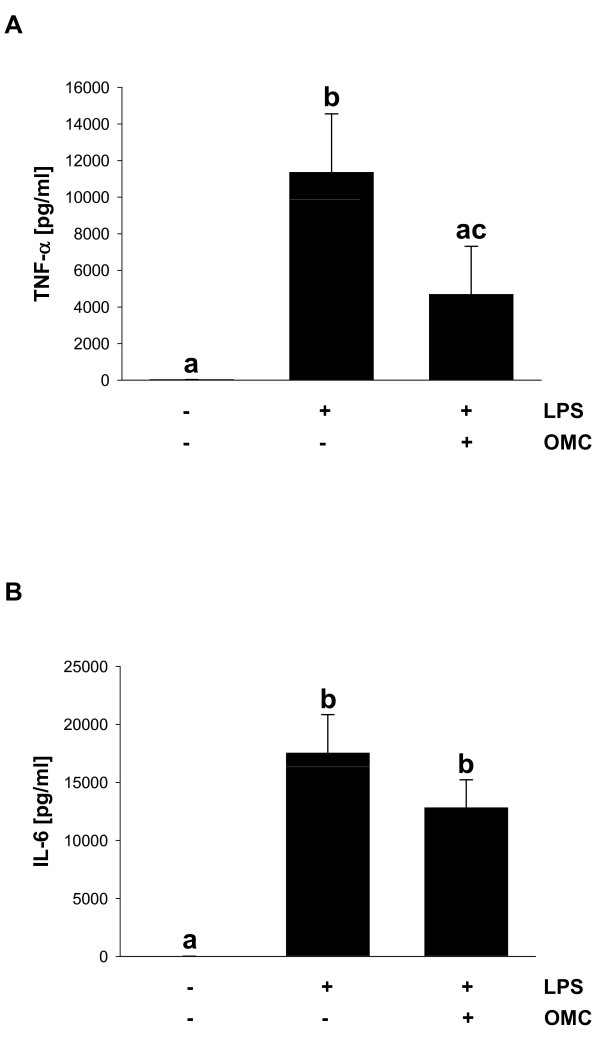
**OMC reduces LPS-induced cytokine production in vivo**. (A) TNF-α and (B) IL-6 were determined in plasma in mice treated with OMC (1000 mg/kg) and LPS (0.1 mg/kg) as described in *Materials and Methods*. The data are means ± S.D, *n *= 3-6. Means without a common letter differ, *P *< 0.05.

To determine whether OMC suppresses inflammatory response *in vivo *we evaluated OMC efficacy in a mouse model of LPS-induced endotoxemia. Results showed that intraperitoneal injection of LPS (0.1 mg/kg) caused the significant elevation of plasma TNF-α at 90 minutes after the LPS challenge, whereas in mice pretreated by oral application of OMC (1000 mg/kg), plasma TNF-α levels decreased by 58.7% (Figure 4A). Moreover, we have found that OMC suppressed the LPS-dependent induction of the plasma IL-6 levels in mice by 26.9% (Figure 4B). Therefore, our data suggest that OMC possesses anti-inflammatory activity *in vivo*.

### OMC possesses immunosuppressive activity

**Figure 5:**
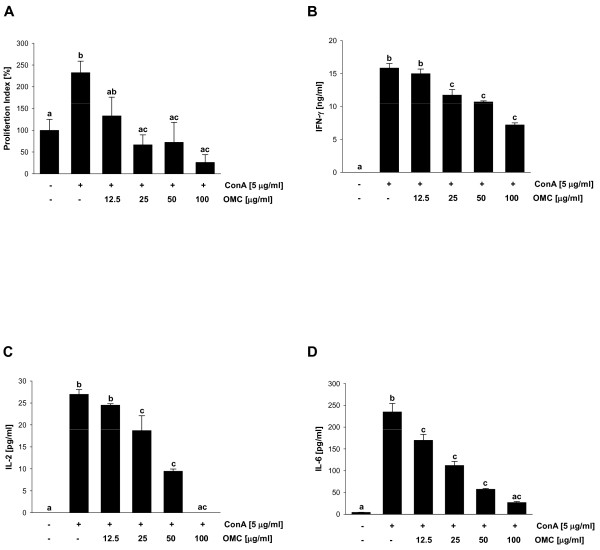
**OMC inhibits ConA-induced splenocyte proliferation and production of IFN-γ, IL-6 and IL-4**. Splenocytes were cultured in the presence of ConA (5 μg/ml) and OMC (0-100 μg/ml) for 72 hours. (A) cell proliferation, (B) production of IFN-γ, (C) IL-2, and (D) IL-4 was determined as described in *Materials and Methods*. The data are means ± S.D. of triplicate measurements. Similar data were obtained in two additional independent experiments. Means without a common letter differ, *P *< 0.05.

To determine the immunosuppressive potential of OMC, we evaluated the effect of OMC on mitogen-stimulated splenocyte proliferation *in vitro*. ConA-dependent induction of splenocyte proliferation was significantly reduced by OMC (Figure 5A), suggesting immunosuppressive activity of OMC. Because the inhibition of splenocyte proliferation could be associated with suppressed production of proinflammatory cytokines by lymphocytes (B-cells), we next evaluated the levels of IFN-γ, IL-2 and IL-6 in splenocytes stimulated with ConA in the presence of OMC. We found that OMC markedly suppressed ConA-dependent production of IFN-γ and IL-2, suggesting that OMC modulates Th1-mediated immune response (Figure 5B and 5C). In addition, OMC suppressed ConA-dependent production of IL-6 in splenocytes (Figure 5D), further confirming anti-inflammatory activity of OMC.

### Analysis of biologically active compounds in OMC

Previous chemical studies on the oyster mushroom indicate that water-soluble polysaccharides [32], amino acids, and nucleotides [33] are its major natural compounds, along with minor low polar sterols, terpenes [34], and vitamins that include water soluble vitamins B and C, and fat-soluble vitamin D [35]. The chemical composition of mushrooms directly depends on their source because different lineages of the same mushroom demonstrated diverse activities [36-38]. Furthermore, growing conditions, time of harvest, processing, and storage conditions are crucial factors that can influence the biological activity of mushrooms and their extracts [39,40]. Acidic and enzymatic hydrolysis of lyophilized oyster mushrooms and OMC was performed as described in *Materials and Methods *and demonstrated that lyophilized oyster mushrooms contain 63.5% and 0.3%, whereas OMC contains 5.6% and 0.3% of β-glucans and α-glucans, respectively. In addition, LC/UV/MS analysis of OMC demonstrated the presence of amino acids isoleucine, leucine, tyrosine, and phenylalanine, and cytidine monophosphate (CMP), adenosine monophosphate (AMP), guanosine monophosphate (GMP) and vitamin B2 (not shown).

## Discussion

In this study, we have evaluated anti-inflammatory activities of the oyster mushroom concentrate (OMC). Here we show that i) OMC markedly suppressed LPS-dependent production of TNF-α, IL-6, and IL-12 in macrophages; ii) OMC inhibited LPS-induced production of PGE_2 _and NO through the downregulation of expression of COX-2 and iNOS in macrophages, respectively; iii) OMC suppressed LPS-dependent activation of AP-1 and NF-κB; iv) OMC inhibited plasma levels of TNF-α and IL-6 in a mouse model of LPS-induced endotoxemia; v) OMC inhibited ConA-induced splenocyte proliferation and production of IFN-γ, IL-2, and IL-6; and vi) chemical analysis demonstrated the presence of α- and β-glucans, isoleucine, leucine, tyrosine, phenylalanine, AMP, CMP, GMP, and vitamin B2 in OMC. The results presented in this study are the first to demonstrate that OMC inhibits the inflammatory response in macrophages, possesses immunosuppressive activity, and inhibits inflammation in mice.

The immunomodulatory effects of mushrooms are usually associated with the stimulation of the immune system by a variety of polysaccharides. These effects include maturation of dendritic cells, stimulation of natural killer (NK) cell activity, and the activation of T and B lymphocytes [18,41]. On the other hand an acidic polysaccharide isolated from *Phellinus linteus *(PL) decreased IL-2, IFN-γ, and TNF-α production in splenocytes [42]. Methanol extract from *Pleurotus florida *demonstrated anti-inflammatory activities *in vivo*; however, the mechanism of its activity was not addressed [28]. Interestingly, Yu *et al* recently demonstrated an induction of the immune response by the stimulation of the TNF-α production in RAW264.7 cells treated with the oyster mushroom *Pleurotus eryngii *[43]. In contrast to Yu *et al*[43] our data with the oyster mushroom *Pleurotus ostreatus *(oyster mushroom concentrate, OMC) demonstrates the opposite effect, inhibition of LPS-induced TNF-α production in RAW264.7 cells treated with OMC. Moreover, OMC demonstrated its anti-inflammatory effect by the inhibition of IL-6, IL-12, PGE_2_, and NO production in RAW264.7 cells. Mechanistically, these effects were mediated by the downregulation of expression of COX-2 and iNOS through the inhibition of transcriptional activity of AP-1 and NF-κB. In addition, OMC also suppressed plasma levels of TNF-α and IL-6 in mice challenged with LPS. Although Yu *et al*[43] did not detect any changes in the ConA-induced secretion of IFN-γ from splenocytes isolated from mice fed with 1% oyster mushroom for 4 weeks, our data demonstrate that OMC suppressed ConA-induced proliferation as well as the secretion of IFN-γ, IL-2, and IL-6 from mouse splenocytes. The opposing results from the Yu *et al* study [43] and our study could be caused by several factors. Although the preparation of the oyster mushrooms in both studies is practically identical (freeze drying of the fresh mushrooms), in our study, we used a different strain of the oyster mushroom, *Pleurotus ostreatus*. In addition, we used a water extract (OMC) where insoluble particles were removed by the filtration, whereas Yu *et al*[43] dissolved freeze-dried oyster mushroom in DMSO and used the whole, unfiltered extract. If, however, the same strain of mushroom had been used, the presence and amount of the biologically active compounds could be different. As mentioned above, the chemical composition of different lineages of the same mushroom could be dissimilar [36-38]. In addition, the conditions of growing, harvesting, processing, and storaging also affect the composition, and, therefore, the biological activity of the mushrooms [39,40].

In our study, we analyzed the chemical composition of OMC, and we identified the water-soluble α- and β-glucans and small organic molecules. Therefore, this analysis could help develop a specific "fingerprint" for the biologically active mushrooms with particular activities. We previously tested the biological activities of *Pleurotus ostreatus *from different sources and identified the mushroom with the highest biological activity, which we then selected for use in our study (unpublished results). A more comprehensive chemical analysis of the OMC and further bioguided fractionation would enable a better understanding of the bioactives.

The anti-inflammatory activity of OMC can be attributed to different compounds. As previously mentioned, OMC contains the amino acids isoleucine, leucine, tyrosine, and phenylalanine. Interestingly, the original study published 25 years ago, demonstrated anti-inflammatory activity of isoleucine and leucine and suggested that this anti-inflammatory activity is related to interference with the action and/or synthesis of prostaglandins [44]. Another compound with anti-inflammatory activity that we identified in OMC is vitamin B2. As recently demonstrated, vitamin B2 suppressed TNF-α, IL-1, IL-6, and NO plasma levels and downregulated expression of iNOS in livers in mice challenged with LPS [45,46]. As previously mentioned, the most abundant compounds in mushrooms are glucans, and their presence is associated with the stimulation of the immune system [18]. However, OMC contains 5.8% of water-soluble glucans (5.56% of β-glucans and 0.26% of α-glucans). Therefore, it is possible that these glucans are responsible for the anti-inflammatory activity of the oyster mushroom. As recently demonstrated, water soluble β-glucans from other edible mushrooms demonstrated anti-inflammatory activity through the inhibition of NO production in activated macrophages (*Collybia dryophila) *[47], the inhibition of leukocyte migration to injured tissues (*Pleurotus pulmonarius) *[26], and the inhibition of edema (*Agaricus blazei) *[48]. In addition, an insoluble β-glucan (pleuran) from *Pleurotus ostreatus *suppressed inflammation in an animal model of colitis [27].

## Conclusions

In conclusion, the results of our study show that the edible oyster mushroom possesses anti-inflammatory activity. As such, the mushroom and its extract or concentrate, such as OMC, can be considered a functional food that has the potential to control inflammation. Although the biological activity is now better understood, we next seek to identify the responsible biologically active compounds. Further studies elucidating the exact mechanism(s) responsible for the anti-inflammatory activity of this culinary mushroom are necessary.

## List of abbreviations

AP-1: activator protein-1; ConA: concanavalin A; COX-2: cyclooxygenase-2; iNOS: inducible nitric oxide synthase; IFN-γ: interferon-γ; IL: interleukin; LPS: lipopolysaccharide; NO: nitric oxide; NF-κB: nuclear factor-κB; OMC: oyster mushroom concentrate; PGE_2_: prostaglandin E2; TNF-α: tumor necrosis factor-α.

## Competing interests

The authors declare that they have no competing interests.

## Authors' contributions

AJ, SD, JS and DS designed research; AJ, SD and QW conducted research; AJ, JS and DS analyzed data; DS wrote the paper and had the primary responsibility for final content. All authors read and approved the final manuscript.
